# Engineering of Interface Barrier in Hybrid MXene/GaN Heterostructures for Schottky Diode Applications

**DOI:** 10.1021/acsami.4c13225

**Published:** 2024-10-18

**Authors:** Dominika Majchrzak, Karol Kulinowski, Wojciech Olszewski, Rafał Kuna, Daria Hlushchenko, Adrianna Piejko, Miłosz Grodzicki, Detlef Hommel, Robert Kudrawiec

**Affiliations:** † Łukasiewicz Research Network - PORT Polish Center for Technology Development, Stabłowicka 147, 54-066 Wrocław, Poland; ‡ Department of Semiconductor Materials Engineering, Wrocław University of Science and Technology, Wyb. Wyspiańskiego 27, 50-370 Wrocław, Poland; § Institute of Experimental Physics, University of Wrocław, Maksa Borna 9, 50-204 Wrocław, Poland; ∥ Department of Nanometrology, Wroclaw University of Science and Technology, Janiszewskiego 11/17, 50-372 Wrocław, Poland; ⊥ Institute of Low Temperature and Structure Research, Polish Academy of Sciences, Okólna 2, 50-422 Wrocław, Poland

**Keywords:** MXene, GaN, interface study, spectroscopy, Schottky diode

## Abstract

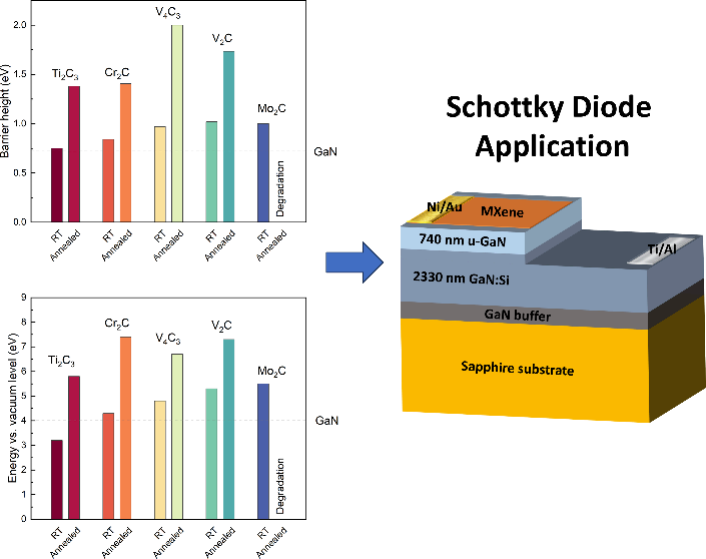

The Fermi level position at the interface of a heterostructure is a critical factor for device functionality, strongly influenced by surface-related phenomena. In this study, contactless electroreflectance (CER) was utilized for the first time to investigate the built-in electric field in MXene/GaN structures with the goal of understanding the carrier transfer across the MXene/GaN interface. Five MXenes with high work functions were examined: Cr_2_C, Mo_2_C, V_2_C, V_4_C_3_, and Ti_3_C_2_. The physicochemical properties of the MXene/GaN structures were analyzed by using X-ray and UV photoelectron spectroscopies. It was shown that upon the coverage of the GaN surface by all investigated MXenes, a shift in the position of the surface Fermi level occurs, consequently raising the interface barrier. Additionally, the physicochemical stability of MXenes on the GaN surface was studied after annealing the structures at 750 °C. Our findings indicate that the annealing process increases the barrier height and the ionization energies of all studied structures. Furthermore, it has been shown that removing excess MXene material from the surface did not significantly impact the built-in electric field, emphasizing the robust physicochemical stability of the MXenes on the GaN surface. To validate the potential of engineering of MXene/GaN interface barrier, Schottky diodes with MXenes exhibiting the highest barrier height (Mo_2_C and V_2_C) were demonstrated.

## Introduction

For over three decades, there has been extensive research on wurtzite GaN-based compounds, resulting in significant advancements in light technology.^1−3^ These semiconductors are widely utilized in the optoelectronics and electronics industry with applications such as light emitters and high-power devices.^4−10^ Despite their utility, the performance of these devices in some cases is limited by the intrinsic properties of GaN-based materials, indicating the need for further enhancement or integration with other materials.

Recently, a new group of 2D materials has emerged, characterized by the standard chemical formula M_*n*+1_X_*n*_T_*x*_ (M is transition metal, X is C and/or N, T represents the surface terminating group like −O, −OH, −F, and *n* = 1–3).^11,12^ These atomically thin transition metal carbides/nitrides were named by MXenes, and to date, over 15 MXenes with various transition metals have been synthesized,^13,14^ with more than 70 predicted to be stable.^15^ MXenes exhibit numerous unique properties, including metallic conductivity, mechanical flexibility, good transmittance, and chemical stability.^16−22^ These properties make them suitable for utilization as electrodes in optoelectronic and electronic devices, particularly given the wide range of work functions (1.6–6.2 eV),^19,23−26^ which offers the MXene group. Furthermore, the van der Waals (vdW) interaction between MXenes and the GaN surface prevents the formation of defects, strains, and atomic disorders typically seen after metal deposition.^27,28^ The features of MXenes help suppress the occurrence of defect states commonly occurring at the metal–semiconductor interfaces.

To date, several articles regarding the combination of MXenes with GaN-based materials have been published.^19,27,29−31^ Luo et al. have shown that the use of MXene electrodes improves the responsivity and reduces dark current of the MXene-GaN-MXene based multiple quantum well photodetector, compared with traditional Metal–Semiconductor–Metal photodetectors (Cr/Au electrodes).^19^ Yi et al. fabricated high-speed photodetectors and stable orange LEDs based on the Ti_3_C_2_T_*x*_/(n/p)-GaN.^29^ Song et al. reported a self-powered, high-performance Ti_3_C_2_T_*x*_ MXene/GaN van der Waals heterojunction-based ultraviolet photodiode.^30^ On the other hand, Wang et al. integrated Ti_3_C_2_T_*x*_ MXene films into GaN HEMTs as the gate contact, showing enhanced gate controllability of the device with an extremely low off-state current (*I*_OFF_), a record high *I*_ON_/*I*_OFF_ current ratio of ≈10^13^ (which is 6 orders of magnitude higher than conventional Ni/Au contact).^31^

Given the potential of enhancing Schottky contact engineering with GaN through MXene integration, we investigated the physical phenomena occurring at the different MXene/GaN interfaces. The Fermi level position at the interface of a heterostructure is a critical parameter for device functionality, as it can be influenced by surface-related phenomena. In this study, we utilized contactless electroreflectance (CER) to examine the built-in electric field in five MXene/GaN structures, aiming to understand carrier transfer across the MXene/GaN interface. For the first time, we determined experimentally the barrier heights at different MXene/GaN interfaces. Additionally, X-ray and UV photoelectron spectroscopies were used to investigate the physicochemical properties of the MXene/GaN structures. To validate the potential of MXene/GaN interface barrier engineering, Schottky diodes with MXenes exhibiting the highest barrier heights (Mo_2_C and V_2_C) were demonstrated. Our findings make a significant contribution to the field of semiconductor heterostructures and their applications in advanced electronic devices. The insights provided by this study could pave the way for enhanced device performance through tailored interface engineering, highlighting types of MXene materials capable of forming Schottky contacts and ohmic contacts with the GaN surface.

## Materials and Methods

### Structure Growth and MXene Transfer

To harness the potential of modulation spectroscopy and to gain a comprehensive understanding of the electronic phenomena occurring at the MXene/GaN interface, structures known as van Hoof GaN were studied.^32−34^ For a typical van Hoof structure, a homogeneous electric field is expected within the undoped GaN layer. The magnitude of this field can be estimated by knowing the thickness of GaN and the Fermi level pinning at both the GaN surface and the GaN/n-GaN interface. Despite the GaN being covered with MXene, electronic passivation of the GaN surface states may still occur, affecting the position of the surface Fermi level.

**Figure 1:**
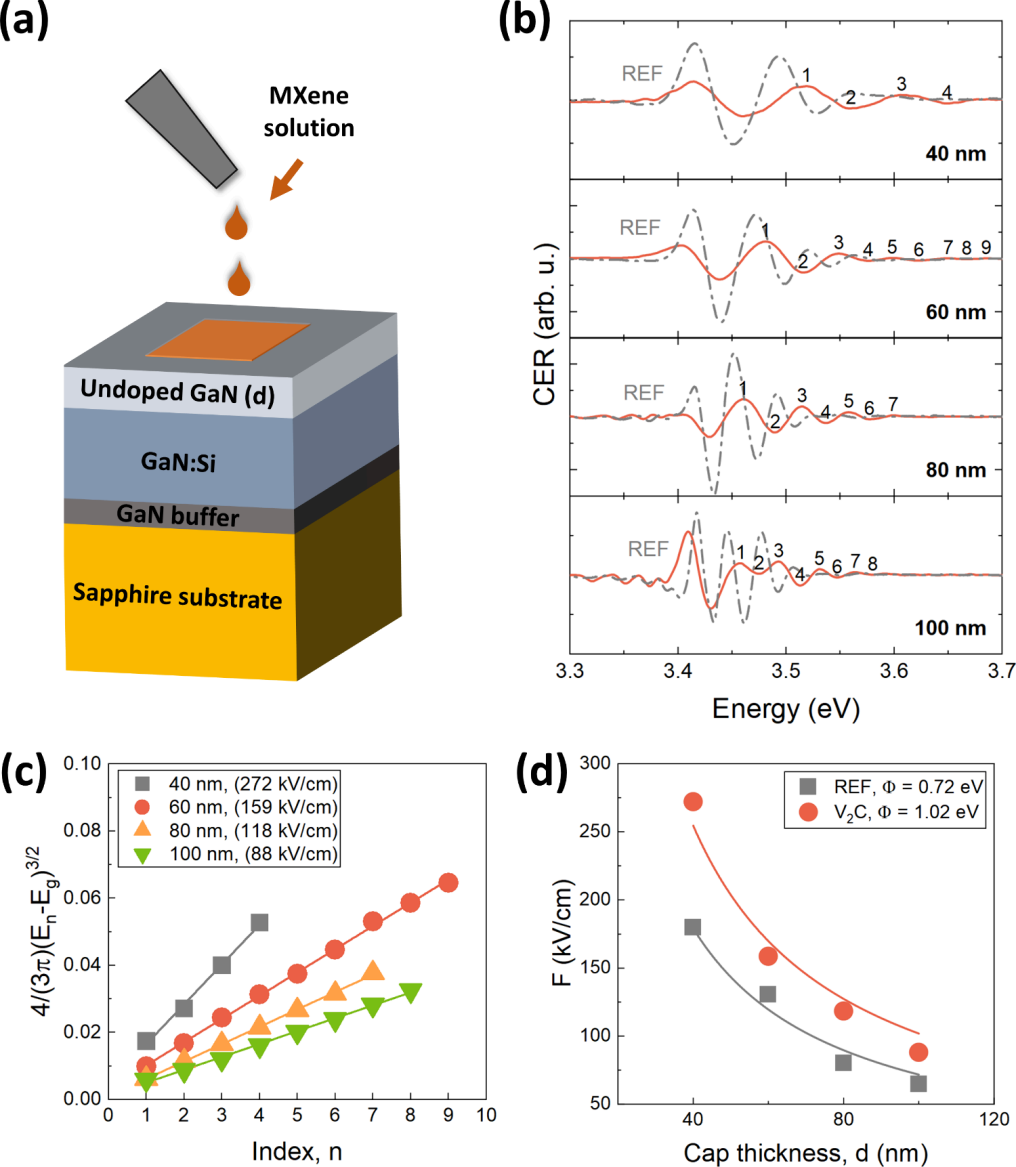
(a) General scheme of the MXene/GaN van Hoof structure. (b) Room temperature CER spectra of reference GaN (gray dashed-dot lines) and V_2_C/GaN van Hoof structures (orange solid lines) with 40, 60, 80 and 100 nm thick cap layers and (c) corresponding analysis of the built-in electric field for reference GaN and V_2_C/GaN structures with different thickness of GaN cap layers. (d) Calculation of the surface barrier height for both GaN and V_2_C/GaN structures together with the fitting curves. Determined values of the built-in electric field and surface barrier height are given in the legends.

All of the GaN van Hoof structures were deposited in CCS3 × 2FT AIXTRON vertical showerhead metalorganic vapor phase epitaxy (MOVPE) reactor on 430 μm thick *c*-plane sapphire substrates with a 0.2° offcut angle. The precursors employed were trimethylgallium and ammonia (NH_3_), with hydrogen (H_2_) serving as the carrier gas. Figure 1a illustrates a schematic of the GaN van Hoof structures coated with MXenes. The structures consisted of a 2.2 μm thick undoped GaN buffer layer, 0.5 μm thick n-type GaN layers doped with silicon using silane (SiH_4_) obtaining a dopant concentration of 5 × 10^18^ cm^–3^, and a cap layer of undoped GaN with thickness of 40, 60, 80, and 100 nm. All layers were grown at a 150 mbar pressure and a temperature of 1045 °C.

The epitaxial GaN van Hoof structures were treated by dipping in HCl solution (35–38%) for 1 min at room temperature, then rinsed with deionized water, and blown dry with N_2_. Subsequently, the commercial MXene flakes in ethanol solution (2D Semiconductors) were applied onto the sample’s surface, allowing it to air-dry naturally. The amount of applied MXene material was controlled by adjusting the solution concentration and maintaining a consistent number of drops across all samples.

### CER Measurements

During CER experiment, the samples were positioned within a custom-designed capacitor as follows: the sample was affixed (using silver paste) to the lower electrode, while the semitransparent upper electrode (constructed from copper wire mesh) was situated 0.5 mm above the sample. An alternating external voltage (280 Hz and 3 kV) facilitated the modulation of band-bending. Laser-driven xenon lamp light was dispersed by a single grating 0.75 m Andor monochromator. The measurements were conducted in a so-called “dark configuration” where the sample was illuminated with monochromatic light while the external modulation remained active. Light reflected from the sample was detected using a lock-in technique, employing a photomultiplier.

### XPS/UPS Measurements

Photoelectron experiments were conducted by utilizing a hemispherical electron energy analyzer (Argus CU) under either UV or X-ray illumination. The employed sources included nonmonochromatic He I (21.21 eV) and monochromatic Al Kα (1486.6 eV). For UPS measurements, photoelectrons were acquired with a 0.01 eV step and a pass energy of 2 eV, while for XPS measurements, a step of 0.1 eV and a pass energy of 20 eV were utilized. The electron collection angle was set at 30°, defined as the angle between the entrance axis of the analyzer and the substrate normal. Binding energy values are referenced to the Fermi level of the electron analyzer, whose position was determined by using a clean reference Ag sample.

### Processing and Electrical Measurements of the MXene/GaN Schottky Diode

A Schottky diode was fabricated using the structure with 2330 nm thick n-GaN layer, doped with silicon using SiH_4_ obtaining a dopant concentration of 2 × 10^18^ cm^–3^, followed by a 740 nm thick undoped GaN cap. First, the mesa area was etched in an ICP-RIE plasma etcher by using a BCl_3_/Cl_2_/Ar mixture. Subsequently, the MXene was transferred onto the remaining undoped GaN surface. Ni/Au and Ti/Al metals were used for the Schottky contact and ohmic contact, respectively. The contacts were deposited using the physical vapor deposition method at a pressure of 3 × 10^–7^ mbar. A reference Schottky diode, without the MXene layer, was prepared by following the same procedure. The room temperature *I*–*V* characteristics for reference GaN, Mo_2_C/GaN, and V_2_C/GaN Schottky diodes were measured by using a Keysight B2901A precision source meter. Each sample was probed with two-terminal transport measurements by using previously prepared metal contacts.

## Results and Discussion

CER spectroscopy represents one of the modulation spectroscopy techniques requiring an external perturbation to modulate a selected parameter within the semiconductor material or structure.^32−34^ In the case of the CER, an external electric field induces band-bending modulation in the near-surface region. When an inherent electric field exists within the structure, it leads to the observation of Franz-Keldysh oscillations (FKO) above the fundamental transition of the studied structure.^32−34^ The period of FKO correlates with the strength of this field. At the doped/undoped GaN interface in the bare van Hoof structure, the Fermi level is positioned close to the conduction band edge (CBE) due to n-type doping. On the opposite side of the undoped layer, the surface Fermi level is pinned by surface states to one of two characteristic surface density of states (SDOS) in the GaN band gap, which result from surface reconstruction and Ga dangling bonds.^35^ The difference in Fermi level positions creates a uniform electric field in the top undoped layer of the van Hoof structure, leading to observable FKO in the CER spectra. Although the GaN is covered with MXene, which might passivate the GaN surface states and affect the surface Fermi level position, analyzing FKO in both bare and MXene-covered van Hoof GaN structures provides insights into the electronic phenomena at the interface. In this study, van Hoof GaN structures with 40, 60, 80, and 100 nm thick undoped cap layer were investigated. Figure 1b shows the room temperature CER spectra for reference GaN (gray dashed-dotted lines) and V_2_C/GaN structures (orange solid lines). All spectra consist of GaN band gap-related resonance (3.43 eV) followed by the FKO. Assuming uniform doping levels across all GaN supports, a decrease in the built-in electric field value is anticipated for thicker cap layers,^36^ which is reflected in a shortened FKO period. Upon comparison of the CER spectra, a noticeable widening of the FKO period is observed for each cap thickness after V_2_C MXene coverage. This indicates an increase in the built-in electric field within V_2_C/GaN induced by V_2_C transfer. The values of the electric field were calculated from the extrema of the FKO period and are depicted in the legend in Figure 1c. The analysis followed the standard procedure outlined by Aspnes and Studna,^33^ where the extremes of FKO are plotted as a function of , resulting in a linear relationship proportional to the built-in electric field *F* under conditions of uniform field. In this context, *E*_*n*_ represents the energy of the *n*-th extremum, *E*_*g*_ denotes the transition energy, φ is the phase, and μ is the electron–hole reduced mass for GaN.

To ascertain the barrier height Φ for GaN and V_2_C/GaN, the electric field’s calculated values were fitted using formula *F* = Φ/*d*, and plotted in Figure 1d. The parameter *d* represents the thickness of the undoped cap layer in the van Hoof structure as a uniform distribution of the electric field is anticipated. The surface barrier height value of 0.72 and 1.02 eV was obtained for GaN and V_2_C/GaN, respectively. The derived GaN value aligns reasonably with reported values for n-type GaN.^32,35,37^ The increase of the surface barrier upon V_2_C coverage of the GaN surface indicates a notable shift in the surface Fermi level position deeper relative to the conduction band minimum of GaN. Furthermore, despite variations in the thickness of the MXene layer across the sample surface, this heterogeneity does not affect the quantitative increase in the potential barrier height. The CER results for V_2_C material applied to the GaN van Hoof structure using one, two, and three drops of solution are shown in Figure S1. As demonstrated, increasing the amount of material on the surface does not significantly impact the height of the potential barrier. The surface barrier height value of 0.7, 1.0, 1.1, and 1.1 eV was obtained for no MXene, 1 drop, 2 drops, and 3 drops of MXene, respectively.

**Figure 2:**
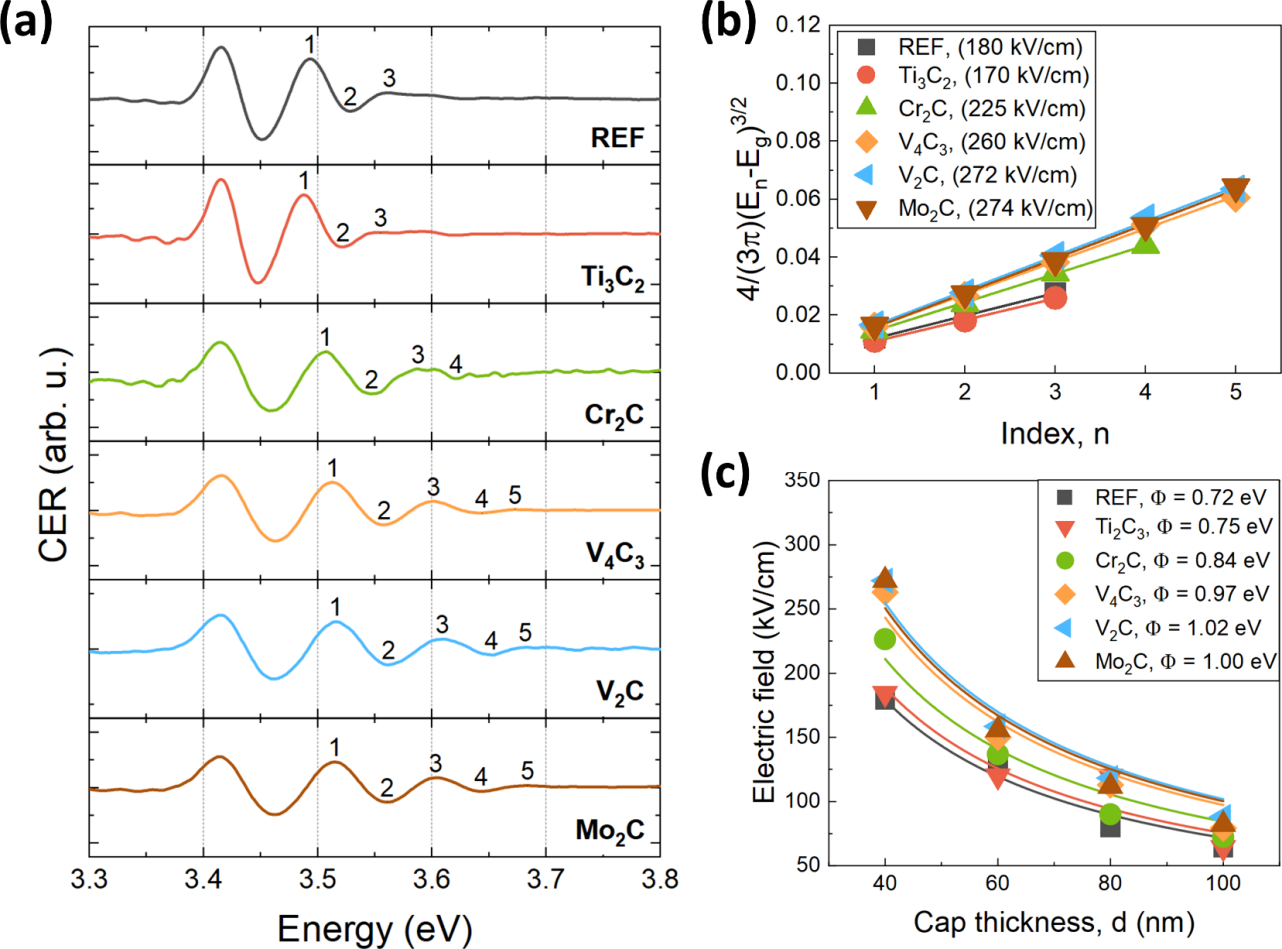
(a) Room temperature CER spectra of reference GaN and different MXene/GaN van Hoof structures with 40 nm thick cap layer. (b) Corresponding analysis of the built-in electric field and (c) calculation of the surface barrier height with the fitting curves for both reference GaN and different MXene/GaN structures. Determined values of the built-in electric field and surface barrier height are given in the legends.

Figure 2a shows the room temperature CER spectra for reference GaN and different MXene/GaN van Hoof structures with 40 nm-thick cap layer. All spectra consist of GaN band gap-related resonance (3.43 eV) followed by FKO. Upon comparison of the CER spectra, a noticeable widening of the FKO period is observed for each MXene coverage. The smallest change in FKO period is observed for Ti_2_C_3_. These findings suggest an increase in the built-in electric field within each MXene/GaN structure induced by MXene transfer. The values of the electric field were calculated from the extrema of the FKO period and depicted in the legend in Figure 2b. The calculation of the surface barrier height for both GaN and different MXene/GaN structures, along with the fitting curves, is illustrated in Figure 2c. It is evident that the smallest change in surface barrier height compared to reference GaN (0.72 eV) is observed for the Ti_2_C_3_ material (0.75 eV). Cr_2_C material coverage results in a higher shift of the barrier height to a value of 0.84 eV. The greatest change can be observed for V_4_C_3_ (0.97 eV), Mo_2_C (1.00 eV), and V_2_C (1.02 eV), respectively. These results suggest that upon the coverage of GaN surface by all investigated MXenes, a shift in the position of the surface Fermi level occurs, consequently raising the interface barrier.

**Figure 3:**
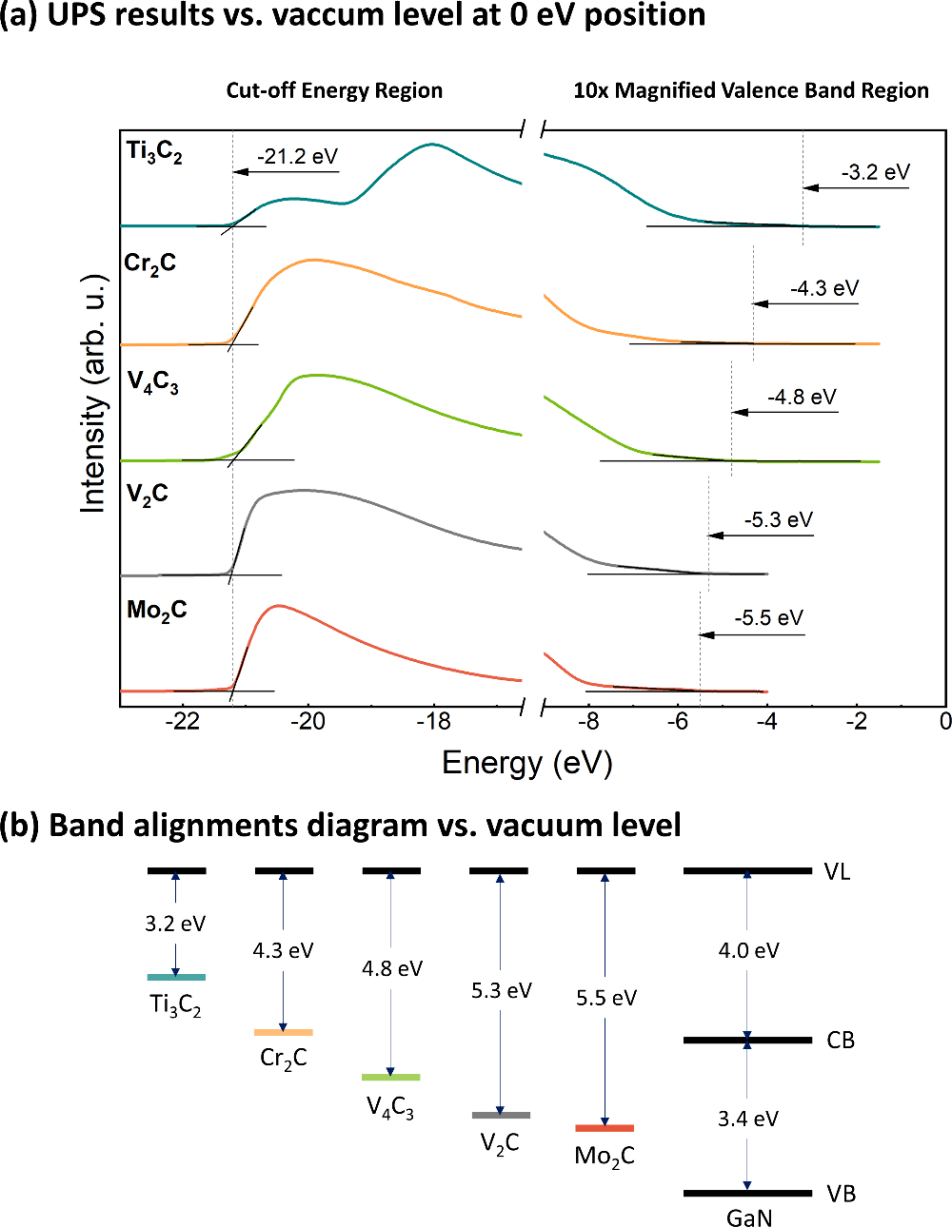
(a) UPS spectra relative to the vacuum level, measured using He I photons (*h*ν = 21.2 eV), and (b) band alignment diagram for all studied MXene/GaN van Hoof structures. Abbreviations: VL - vacuum level, CB - conduction band, VB - valence band.

Figure 3a shows UPS spectra of all studied MXene/GaN van Hoof structures measured using He I photons (*h*ν = 21.2 eV) referenced to the vacuum level (VL). Consequently, the cut-off energy (*E*_cut-off_) for all specimens was set at 21.2 eV, whereas the ionization energy can be determined by extrapolating the onset of spectrum linearly and identifying its intersection with the background in the valence band region. For MXene materials, which exhibit metallic characteristics, the ionization energy coincides with the value of the electron affinity and work function. The lowest ionization energy of 3.2 eV was observed for the van Hoof structure covered with Ti_2_C_3_. Subsequently, values of 4.3 and 4.8 eV were determined for the Cr_2_C and V_4_C_3_, respectively. The highest values of ionization energy were observed for V_2_C and Mo_2_C coverage equal to 5.3 and 5.5 eV, respectively. These high values of determined ionization energy can be attributed to the -O functional group.^24^ The ionization energy for the undoped GaN sample was found to be 7.4 eV, while the electron affinity equal to 4.0 eV (see Figure S2). The determined parameters allow us to construct band diagrams for all studied samples, as presented in Figure 3b, where the ionization energies for all materials refer to the same position of vacuum level. These results suggest that Ti_2_C_3_ emerges as a prospective material for forming Schottky contact to p-GaN, whereas V_2_C and Mo_2_C show potential for creating Schottky contact to n-GaN. Conversely, Ti_2_C_3_ holds promise as a material for achieving ohmic contact to n-GaN, while V_2_C and Mo_2_C may serve for establishing ohmic contact to p-GaN. An important advantage of the presented approach in modifying the Schottky barrier using MXene is the simplicity of the process, specifically the method of depositing the MXene layer on GaN on a macro scale. Another interesting avenue for research could be the mechanical exfoliation of MXene layers on GaN, which would provide a more defined MXene-GaN interface for simulations or calculations. Further research in this area is well justified. As the next step, ab initio density functional theory (DFT) calculations on charge transfer processes across MXene/GaN interfaces are planned, similar to the approach presented in ref.^27^

**Figure 4:**
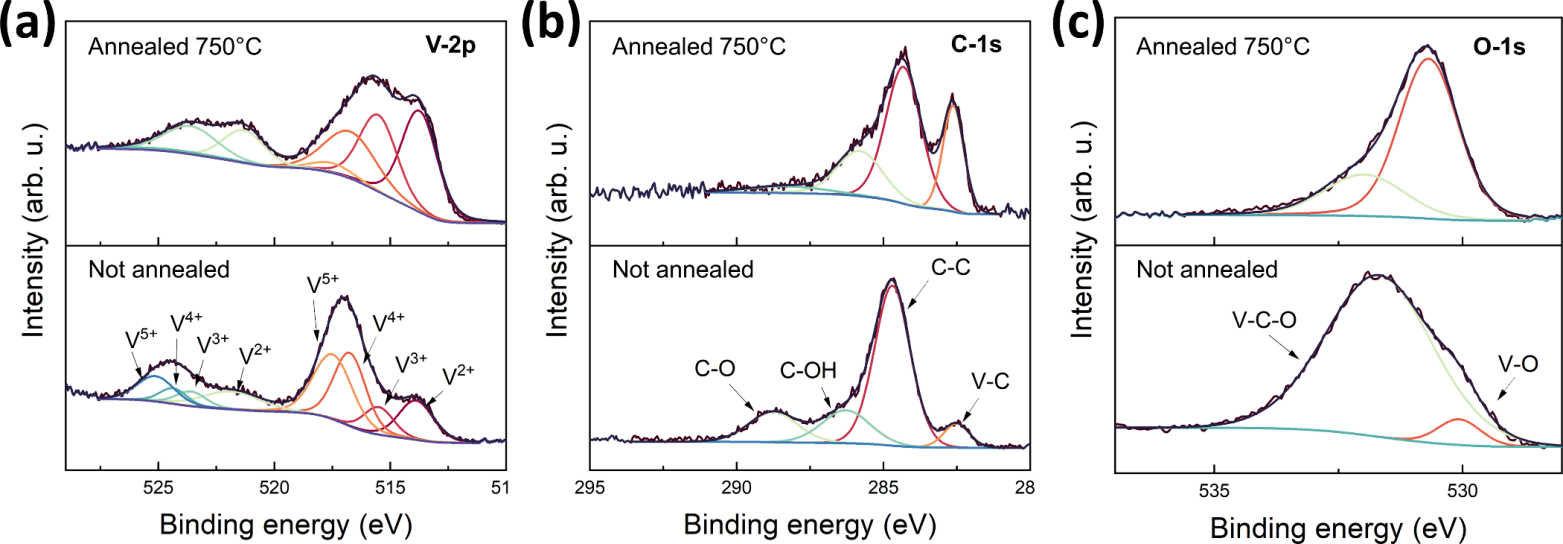
XPS spectra for (a) V-2p, (b) C-1s and (c) O-1s core level lines for not annealed and annealed at 750 °C V_2_C/GaN van Hoof structures.

Figure 4 shows XPS spectra for V-2p, C-1s and O-1s core level lines for V_2_C/GaN van Hoof structure before and after annealing at 750 °C. In Figure 4a, eight fitting peaks observed at 525.1 and 517.5 eV, 524.4 and 516.7 eV, 523.6 and 515.5 eV, and 521.8 and 513.9 eV in the V-2p spectrum are attributed to V^5+^, V^4+^, V^3+^, and V^2+^, respectively. The not annealed V_2_C/p-GaN sample shows a higher proportion of V^5+^ and V^4+^. However, after the sample is annealed at elevated temperatures, the proportion of V^3+^ and V^2+^ becomes more dominant. In the C-1s spectrum, Figure 4b, four fitting peaks at 288.8, 286.3, 284.7, and 282.5 eV correspond to C–O, C–OH, C–C, and V–C bonds, respectively. As observed, annealing the sample results in a relative increase in the intensity of the V–C component, while the intensity from the C–O bond decreases. The O-1s spectrum, Figure 4c, shows two distinct fitting peaks at 531.7 and 530.1 eV, associated with V–C–O and V–O bonds, respectively. In this case, annealing the sample leads to a relative increase in the intensity of the V–O component, while the intensity of the V–C–O bond decreases.

The similar effects can be observed for other studied MXene/GaN van Hoof structures (see XPS spectra presented in Figure S3 and Figure S4). Only in the case of the Mo_2_C/GaN structure, the degradation of Mo_2_C material at 750 °C was observed.

**Figure 5:**
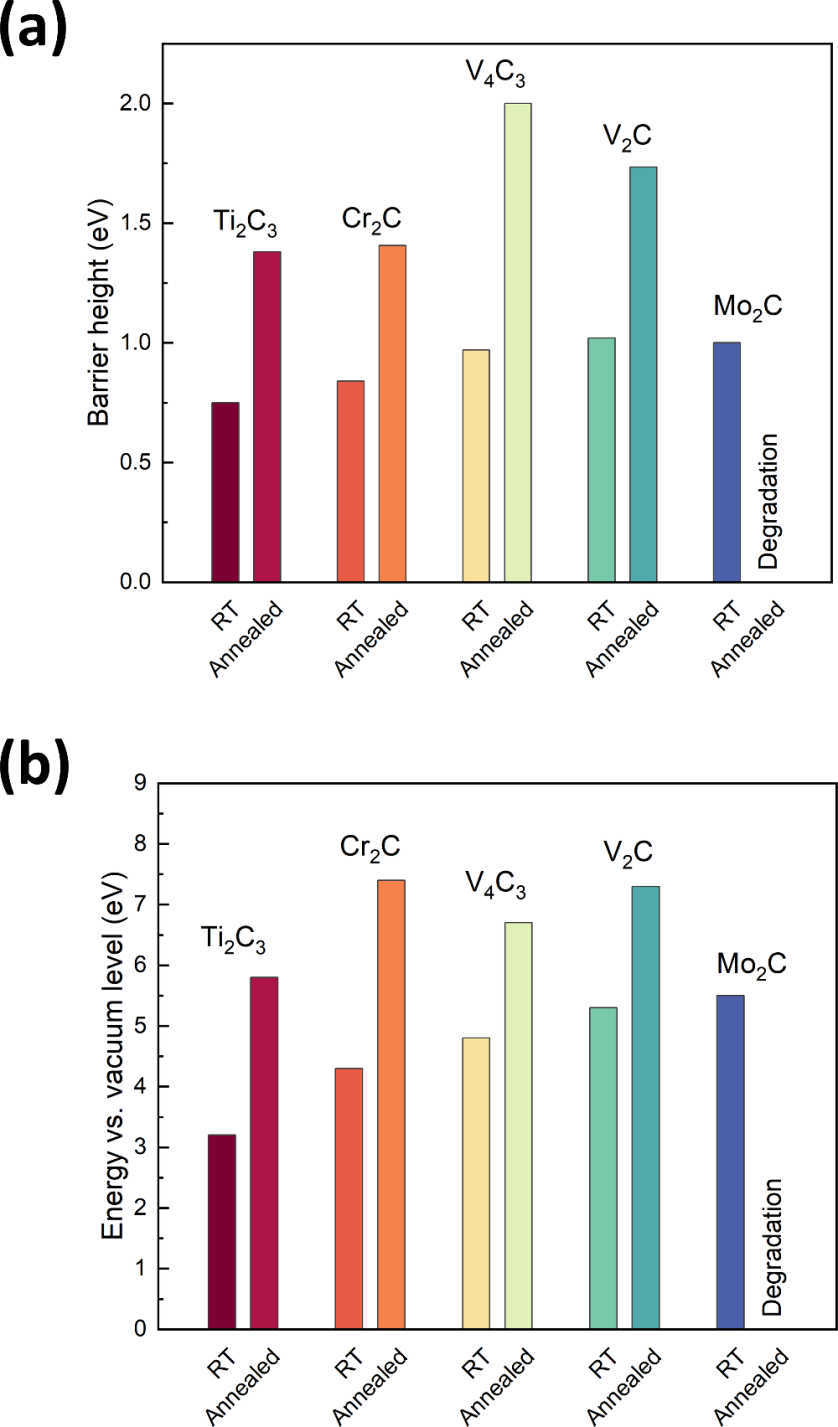
Summary of (a) barrier heights derived from CER measurements and (b) ionization energies from UPS analysis for all examined MXene/GaN samples, both before and after annealing at 750 °C.

Due to the significant structural changes that the MXenes undergo when subjected to high temperatures, necessary during the processing of electrical contacts, the MXene/GaN interface was also examined after annealing at 750 °C using CER and UPS techniques. In Figure 5 presents a summary of (a) barrier heights derived from CER measurements and (b) ionization energies from UPS analysis for all examined MXene/GaN samples, both before and after annealing at 750 °C. As can be observed, the annealing process increases the barrier height and the ionization energies of all studied structures. In the case of the Mo_2_C/GaN structure, significant degradation of the Mo_2_C material was observed at 750 °C; therefore, CER/UPS results were not presented. The results of UPS analysis and CER measurements for all studied MXene/GaN van Hoof structures after annealing at 750 °C can be found in Figure S5 and Figure S6, respectively.

**Figure 6:**
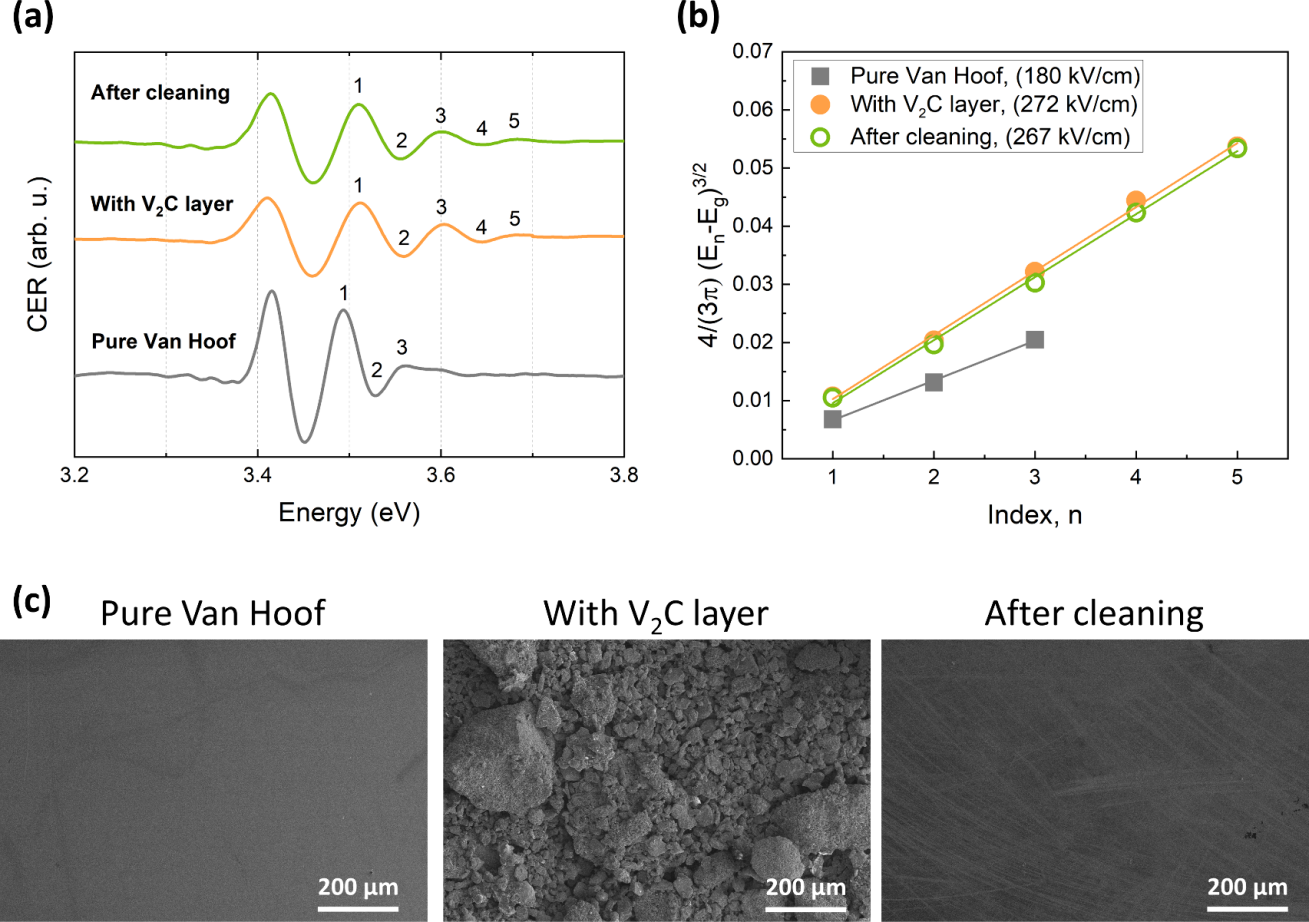
(a) Room temperature CER spectra, (b) analysis of the built-in electric field, and (c) corresponding SEM images for reference GaN and V_2_C/GaN van Hoof structures with 40 nm thick cap layer. “After cleaning” refers to V_2_C/GaN van Hoof structure after acetone treatment and wiping of the surface. Extracted values of the built-in electric field are given in the legend.

To study physicochemical stability of MXenes on the GaN surface, van Hoof GaN structures with 40 nm thick cap layer were used. Figure 6a shows the room temperature CER spectra for reference GaN and V_2_C/GaN van Hoof structures before and after cleaning, i.e., acetone treatment and wiping of the surface. All spectra consist of GaN band gap-related resonance (3.43 eV) followed by the FKO. The values of the electric field were calculated from the FKO period and depicted in the legend in Figure 6b. The increase in the built-in field values for V_2_C/GaN structures indicates an increase in the surface barrier height for electrons. The surface barrier height value of 0.72 and 1.02 eV was obtained for GaN and V_2_C/GaN, respectively. As can be seen, removing excess MXene material from the surface did not significantly affect the FKO period or the built-in electric field. The determined surface barrier height for this sample is 1.00 eV. Figure 6c shows the corresponding SEM images for all of the studied van Hoof structures. It is evident that even after the treatment procedure described above, a thin film of MXene material remains on the sample’s surface. These results underscore the high physicochemical stability of V_2_C MXene on the GaN surface and highlight its potential for further investigations and applications in GaN-based devices.

**Figure 7:**
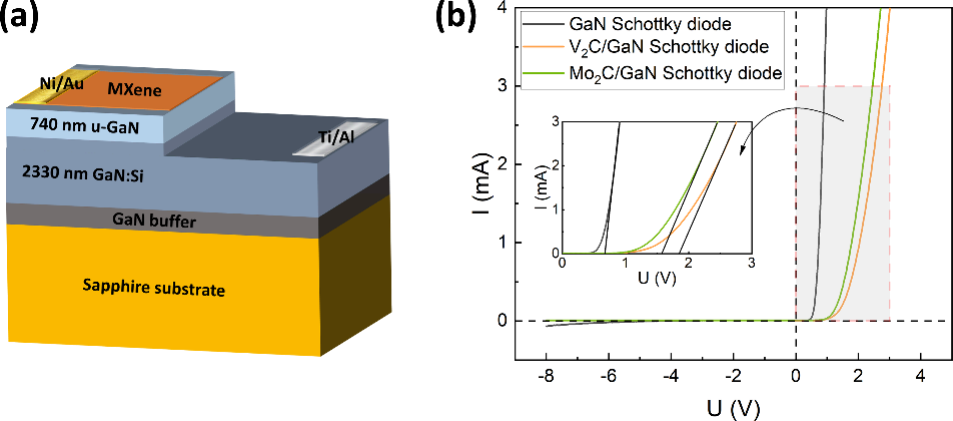
(a) MXene/GaN Schottky diode structure scheme, (b) room temperature *I*–*V* characteristics of reference GaN as well as Mo_2_C/GaN and V_2_C/GaN Schottky diodes. The inset shows a magnified view of the forward voltage region.

To validate the CER/UPS results of the MXene/GaN interfaces, Schottky diodes with MXenes exhibiting the highest barrier height (Mo_2_C and V_2_C) were fabricated. Figure 7a presents the structure scheme of the MXene/GaN Schottky diode. Figure 7b shows the room temperature *I*–*V* characteristics of the studied structures. As observed from the *I*–*V* characteristics, transferring Mo_2_C as well as V_2_C onto the GaN surface increases the forward voltage and reduces the reverse current, indicating an increased potential barrier. All *I*–*V* characteristics are nonlinear, asymmetric with small leakage current of 23 × 10^–6^, 8 × 10^–6^, and 7 × 10^–6^ A for the bare GaN, Mo_2_C/GaN, and V_2_C/GaN structures, respectively, at reverse bias voltage of 6 V. The saturation current *I*_*s*_, series resistance *R*_*s*_, and a value of ideality factor *n* are determined from interpolation of the equation, which describes the current *I* as a function of the applied voltage *V* in the forward bias region: . The value of saturation current is found to be 7.1 × 10^–7^, 9.1 × 10^–7^, and 1.4 × 10^–6^ A for the bare GaN, Mo_2_C/GaN, and V_2_C/GaN structures, respectively. These values closely align with the typical range observed for Schottky diodes. The series resistance was determined to be 49.6, 159.1, and 149.2 Ω for the bare GaN, Mo_2_C/GaN, and V_2_C/GaN structures, respectively. These results indicate very low parasitic series resistance in the studied systems. The value of the ideality factor is equal to 3.7, 9.6, and 11.8 for the bare GaN, Mo_2_C/GaN, and V_2_C/GaN structures, respectively. An ideality factor greater than unity can be attributed to the recombination of electrons and holes in the depletion region. It is also associated with Fermi-level pinning at the interface or with significant voltage drops in the interface region. The high values of *n* observed for the MXene/GaN diodes are consistent with those reported for other van der Waals/covalent crystal interfaces and can be attributed to interfacial disorder.^34,38−40^ The rectification ratio (RR) is determined as the ratio of the forward current to reverse current at a certain applied voltage, and the results are presented in Figure S7. The value of RR is found to be an order of 10^3^ for GaN Schottky diode structure and an order of 10^2^ for MXene/GaN Schottky diodes at a voltage of 2 V. The barrier height was estimated by extrapolating the linear part of the forward current region to the voltage axis, yielding values of 0.66, 1.56, and 1.85 eV for the bare GaN, Mo_2_C/GaN, and V_2_C/GaN structures, respectively. Our findings demonstrate the potential for barrier height engineering at the metallic contact/MXene/GaN interface through the integration of different MXenes with GaN within the same metallization. The presented devices can function as low reverse current Schottky diodes suitable for use in switching power supplies and automotive applications.

## Conclusions

In this study, contactless electroreflectance was utilized for the first time to investigate the built-in electric field in MXene/GaN structures, aiming to understand carrier transfer across the MXene/GaN interface. We examined five MXenes with high work functions: Cr_2_C, Mo_2_C, V_2_C, V_4_C_3_, and Ti_3_C_2_. The physicochemical properties of the MXene/GaN structures were analyzed using X-ray and UV photoelectron spectroscopies. The CER results indicate that covering the GaN surface with any of the investigated MXenes increases the surface barrier. Additionally, we studied the physicochemical stability of MXenes on the GaN surface after annealing the structures at 750 °C. Our findings indicate that annealing increases the barrier height and ionization energies of all of the studied structures. Furthermore, we demonstrated that removing excess MXene material from the surface did not significantly affect the built-in electric field, highlighting the strong physicochemical stability of MXenes on the GaN surface. We emphasized the potential of barrier height engineering and its application in advanced electronic devices through the demonstration of MXene/GaN Schottky diodes with MXenes exhibiting the highest barrier heights (Mo_2_C and V_2_C). The *I*–*V* measurement indicated that transferring MXene onto the GaN surface increases the forward voltage and reduces the reverse current, indicating an increased potential barrier. These results clearly demonstrate that MXenes can be used to engineer the barrier height in Schottky diodes.

## Supporting Information Available

CER results for V_2_C material applied to GaN surface using different amount of MXene/ethanol solution; UPS results for undoped GaN; XPS spectra for all MXene/GaN structures before and after annealing; UPS and CER results for MXene/GaN structures after annealing; Rectification ratio plot for studied Schottky diodes (PDF)

## Abbreviations

CB: conduction band
CER: contactless electroreflectance
FKO: Franz–Keldysh oscillations
HEMT: high-electron mobility transistor
ICP-RIE: inductively coupled plasma-reactive ion etching
MOVPE: metal–organic vapor phase epitaxy
UPS: ultraviolet photoelectron spectroscopy
VB: valence band, VL, vacuum level
XPS: X-ray photoelectron spectroscopy
