# Supplementary material for: Engineering of Interface Barrier in Hybrid MXene/GaN Heterostructures for Schottky Diode Applications
Source: ACS Appl Mater Interfaces. 2024 Oct 18;16(43):59567–75. doi: 10.1021/acsami.4c13225 (PMC11533156; doi:10.1021/acsami.4c13225)
Supplement: Supplementary file 1 — am4c13225_si_001.pdf [file am4c13225_si_001.pdf]

Supporting information

Engineering of Interface Barrier in Hybrid MXene/GaN Heterostructures for Schottky Diode Applications

Dominika Majchrzak [a,*], Karol Kulinowski [b], Wojciech Olszewski [a,c], Rafał Kuna [a], Daria Hlushchenko [a,b], Adrianna Piejko [a,d], Miłosz Grodzicki [a,b], Detlef Hommel [a,e], and Robert Kudrawiec [a,b]

*Corresponding Author; E-mail: Dominika.Majchrzak@port.lukasiewicz.gov.pl

[a] Łukasiewicz Research Network - PORT Polish Center for Technology Development, Stabłowicka 147, 54-066 Wrocław, Poland

[b] Department of Semiconductor Materials Engineering, Wrocław University of Science and Technology, Wyb. Wyspiańskiego 27, 50-370 Wrocław, Poland

[c] Institute of Experimental Physics, University of Wrocław, Maksa Borna 9, 50-204 Wrocław, Poland

[d] Department of Nanometrology, Wroclaw University of Science and Technology, Janiszewskiego 11/17, 50-372 Wrocław, Poland

[e] Institute of Low Temperature and Structure Research, Polish Academy of Sciences, Okólna 2, 50-422 Wrocław, Poland

1. CER results for V_2C material applied to GaN surface using different amount of MXene/ethanol solution

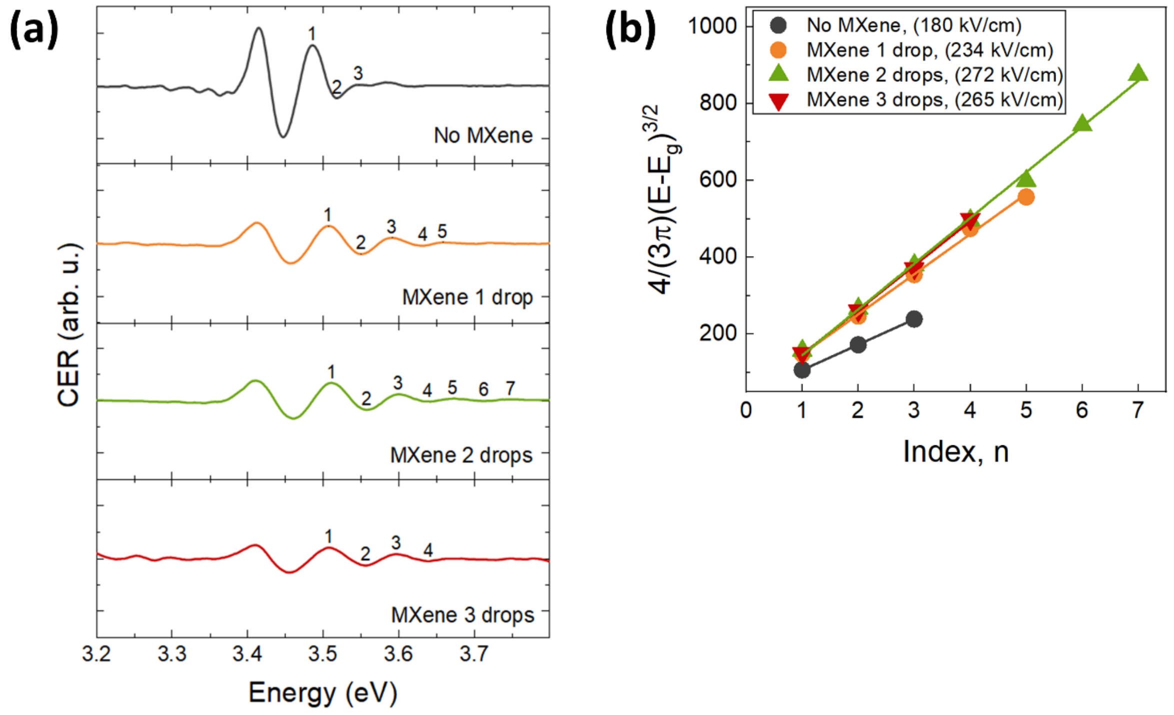

Figure s1. (a) Room temperature CER spectra, (b) analysis of the built-in electric field for V_2C material applied to GaN van Hoof structure with 40-nm-thick cap layer using one, two, and three drops of MXene/ethanol solution.

2. UPS spectrum for undoped GaN

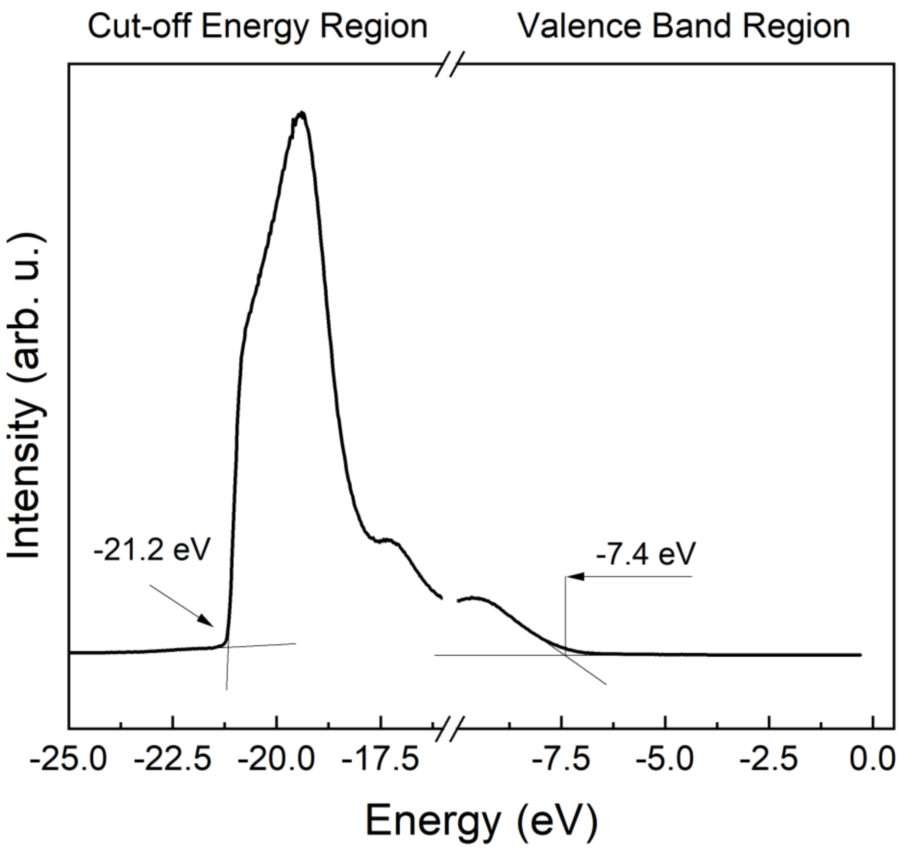

Figure s2. UPS spectrum relative to the vacuum level, measured using He I photons (hν = 21.2 eV), for undoped GaN. The graph reveals that the ionization energy is 7.4 eV, indicating the electron affinity of 4.0 eV.

3. XPS spectra for all MXene/GaN structures before annealing

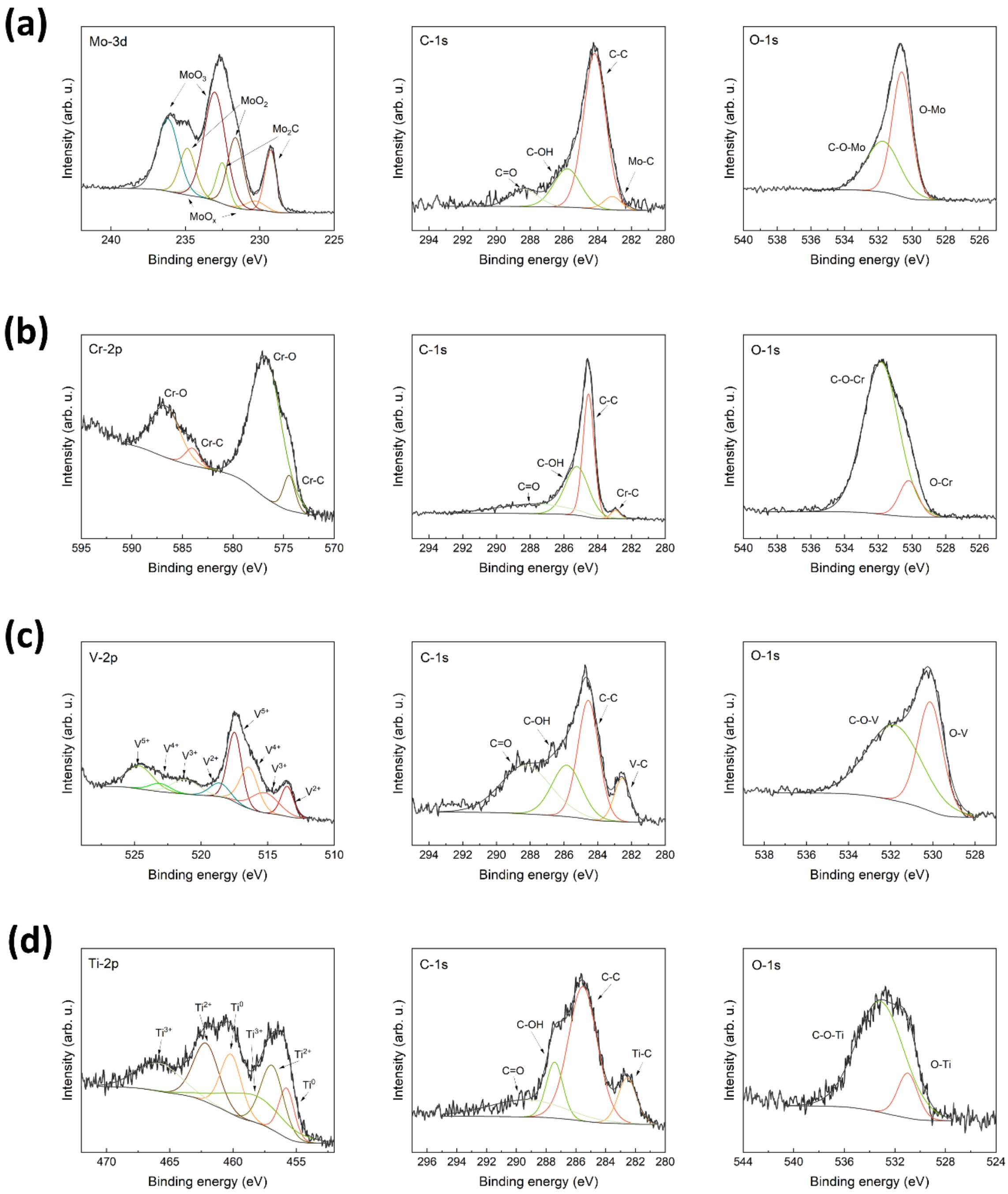

Figure s3. XPS spectra of the main core level lines for (a) Mo_2C/GaN, (b) Cr_2C/GaN, (c) V_4C_3/GaN, (d) Ti_2C_3/GaN van Hoof structures before annealing.

4. XPS spectra for all MXene/GaN structures after annealing

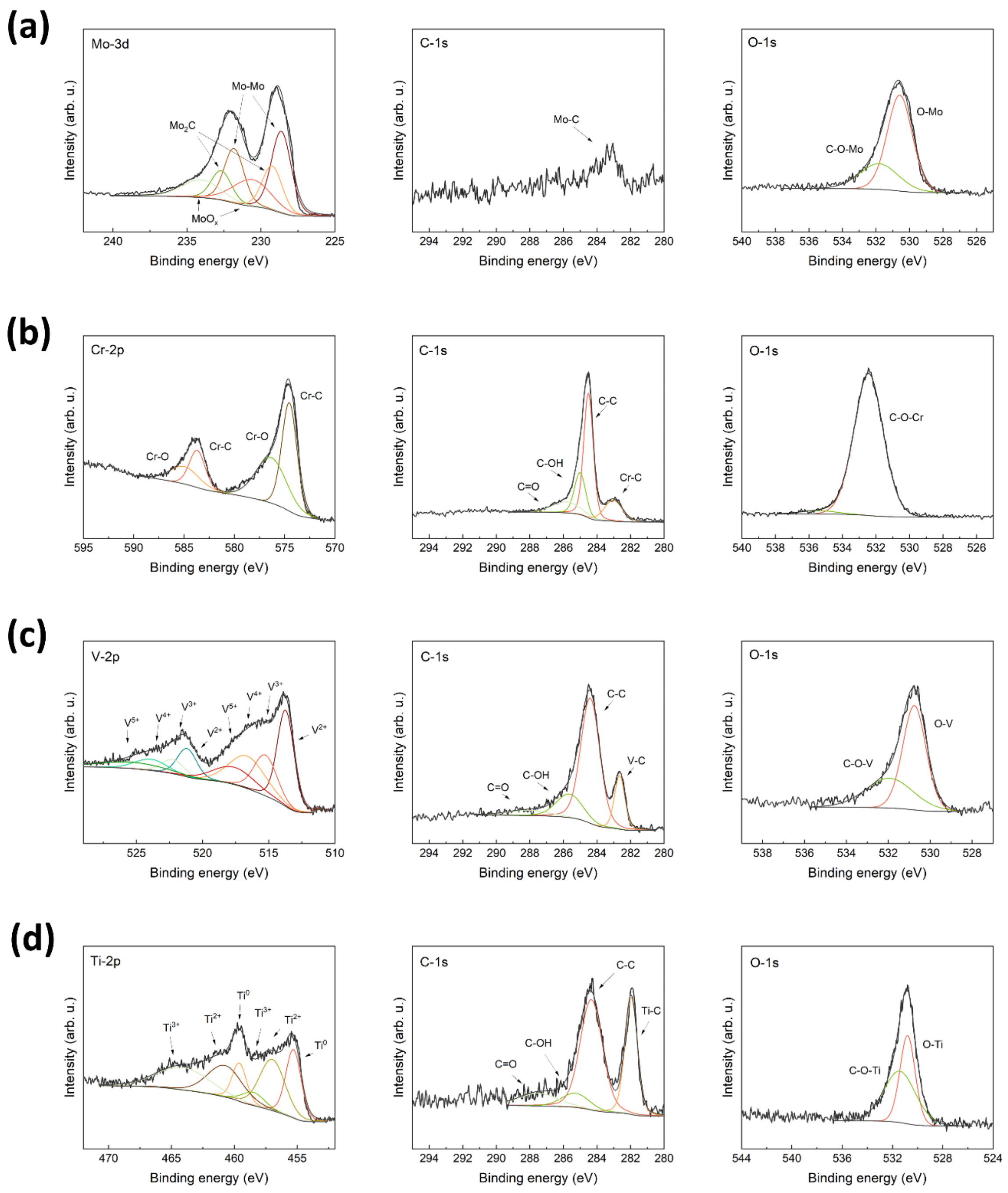

Figure s4. XPS spectra of the main core level lines for (a) Mo_2C/GaN, (b) Cr_2C/GaN, (c) V_4C_3/GaN, (d) Ti_2C_3/GaN van Hoof structures after annealing at 750°C.

5. UPS spectra for annealed MXene/GaN structures

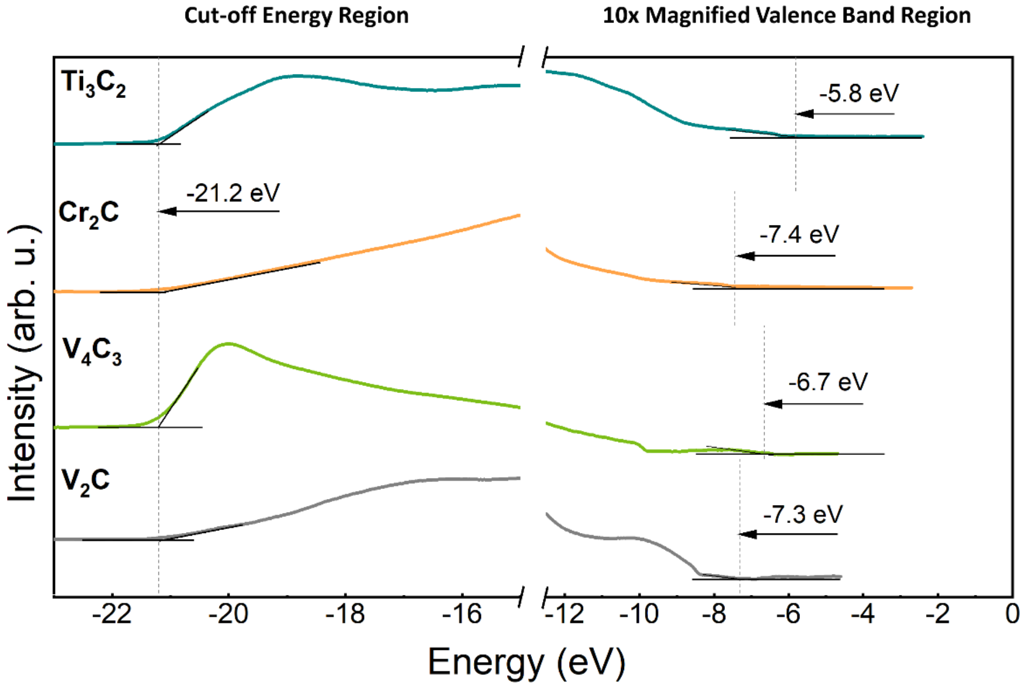

Figure s5. UPS spectra relative to the vacuum level, measured using He I photons (hν = 21.2 eV), for all studied MXene/GaN van Hoof structures after annealing at 750°C.

6. CER results for annealed MXene/GaN structures

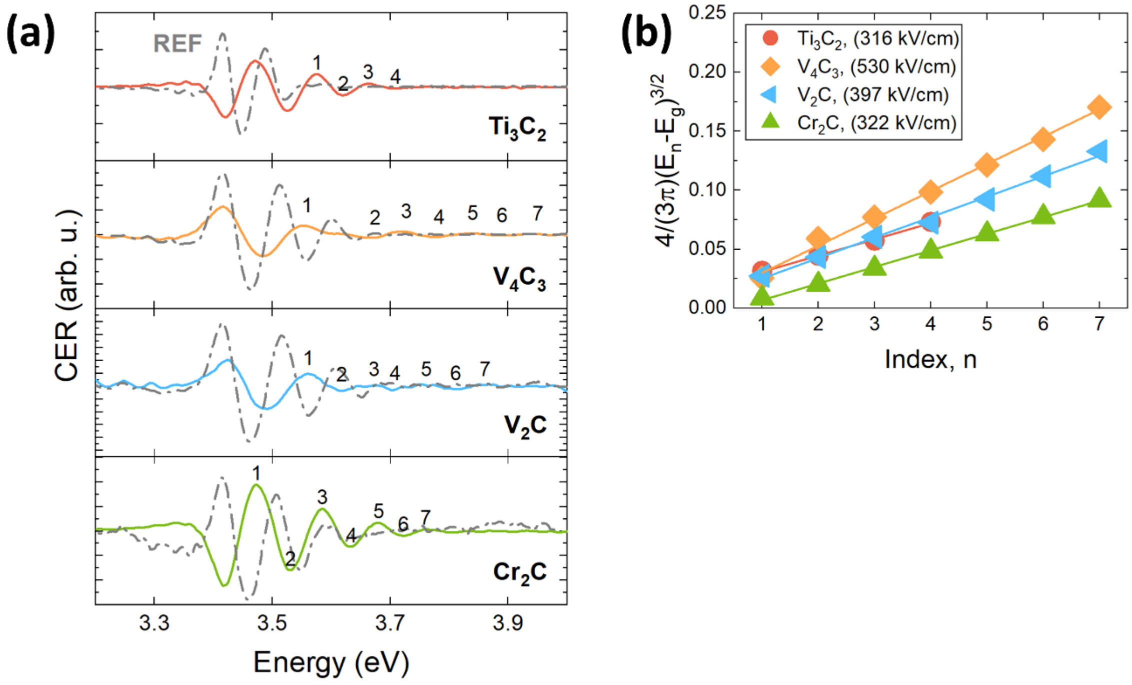

Figure s6. (a) Room temperature CER spectra, (b) analysis of the built-in electric field for different MXene/GaN van Hoof structures with 40-nm-thick cap layer after annealing at 750°C. The gray dashed-dot lines in (a) represent the CER measurements conducted before annealing.

7. Rectification ratio plot for studied Schottky diodes

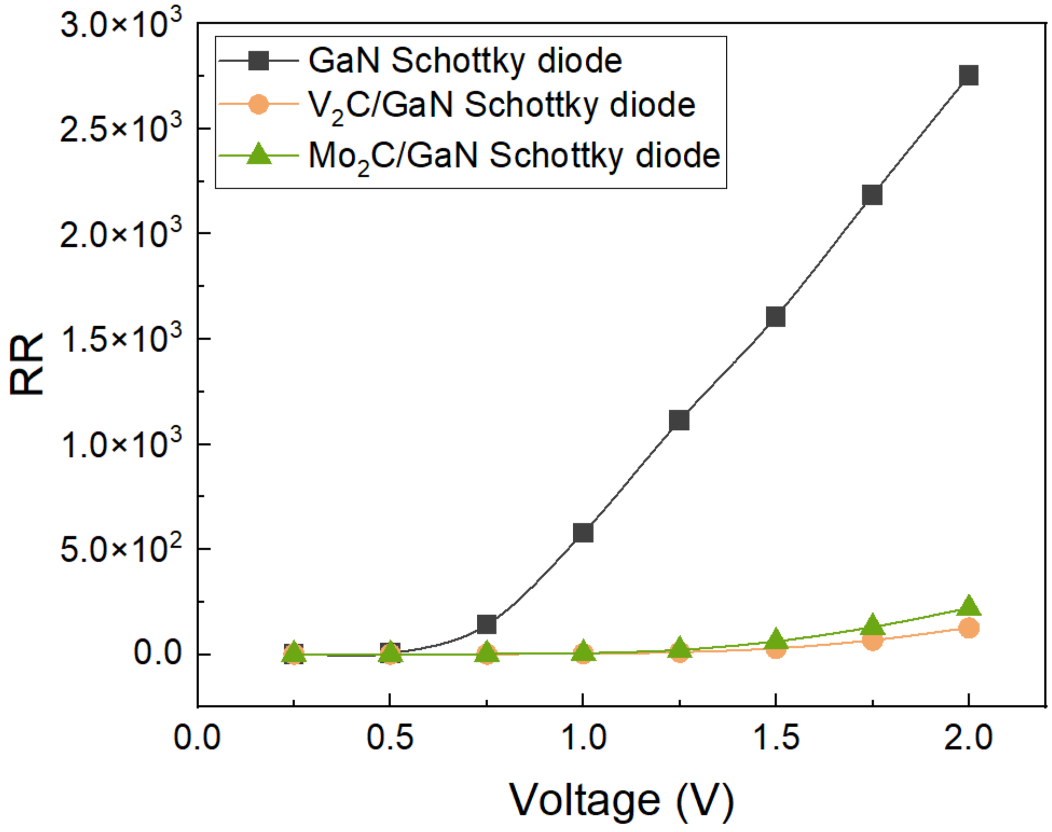

Figure s7. The rectification ratio plot of reference GaN as well as Mo_2C/GaN and V_2C/GaN Schottky diodes.
